# Alternate-Day High-Fat Diet Induces an Increase in Mitochondrial Enzyme Activities and Protein Content in Rat Skeletal Muscle

**DOI:** 10.3390/nu8040203

**Published:** 2016-04-06

**Authors:** Xi Li, Kazuhiko Higashida, Takuji Kawamura, Mitsuru Higuchi

**Affiliations:** 1 Graduate School of Sport Sciences, Waseda University, 2-579-15, Mikajima, Tokorozawa city, Saitama 359-1192, Japan; linokoto@akane.waseda.jp (X.L.); takuji@toki.waseda.jp (T.K.); 2 Faculty of Sport Sciences, Waseda University, 2-579-15, Mikajima, Tokorozawa city, Saitama 359-1192, Japan; mhiguchi@waseda.jp; 3 Institute of Advanced Active Aging Research, Waseda University, 2-579-15, Mikajima, Tokorozawa city, Saitama 359-1192, Japan; 4 Department of Food Science and Nutrition, The University of Shiga Prefecture, 2500 Hassaka-Cho, Hikone city, Shiga 522-8533, Japan

**Keywords:** high-fat diet, alternate-day, mitochondria, skeletal muscle, rat

## Abstract

Long-term high-fat diet increases muscle mitochondrial enzyme activity and endurance performance. However, excessive calorie intake causes intra-abdominal fat accumulation and metabolic syndrome. The purpose of this study was to investigate the effect of an alternating day high-fat diet on muscle mitochondrial enzyme activities, protein content, and intra-abdominal fat mass in rats. Male Wistar rats were given a standard chow diet (CON), high-fat diet (HFD), or alternate-day high-fat diet (ALT) for 4 weeks. Rats in the ALT group were fed a high-fat diet and standard chow every other day for 4 weeks. After the dietary intervention, mitochondrial enzyme activities and protein content in skeletal muscle were measured. Although body weight did not differ among groups, the epididymal fat mass in the HFD group was higher than those of the CON and ALT groups. Citrate synthase and beta-hydroxyacyl CoA dehydrogenase activities in the plantaris muscle of rats in HFD and ALT were significantly higher than that in CON rats, whereas there was no difference between HFD and ALT groups. No significant difference was observed in muscle glycogen concentration or glucose transporter-4 protein content among the three groups. These results suggest that an alternate-day high-fat diet induces increases in mitochondrial enzyme activities and protein content in rat skeletal muscle without intra-abdominal fat accumulation.

## 1. Introduction

Endurance exercise training induces an increase in mitochondrial content in skeletal muscle [1], resulting in increased capacity of muscles to regenerate ATP. The increase in muscle mitochondrial content also results in a change in substrate utilization—with increased fat oxidation and decreased utilization of muscle glycogen [2,3]. Since the performance of endurance exercise is directly related to the muscle glycogen concentration prior to exercise, these biochemical adaptations of skeletal muscle lead to enhanced exercise performance after exercise training.

Aforementioned muscle adaptation is also caused by a high-fat diet feeding. Miller *et al.* [4] demonstrated that a 5-week high-fat diet in rats elevated mitochondrial enzyme activities in skeletal muscle. This biochemical adaptation in skeletal muscle has been reported by other groups in rodents and human subjects [5,6,7,8], although other groups reported opposite results, which high-fat diet feeding results in down-regulation of mitochondrial genes [9] or skeletal muscle from over-feeding-induced obese subject has impaired mitochondrial oxidative capacity [10,11]. Interestingly, in contrast to exercise training [12], the biochemical adaptation to high-fat diet in skeletal muscle occurs slowly, over at least 3–4 weeks [13]. Recent studies have shown the possible mechanisms by which a high-fat diet induces an increase in mitochondrial biogenesis in skeletal muscle [13,14,15,16], e.g., peroxisome proliferator activated receptor (PPAR) δ activation by raising plasma free fatty acids (FFA) and induction of PPAR γ coactivator-1α (PGC-1α).

It is well known that a long-term high-fat diet causes intra-abdominal fat accumulation, insulin resistance, and obesity. Miller *et al.* [4] reported that high-fat diet-fed rats gained more body weight than did the control diet-fed rats, despite a significant increase in mitochondrial enzyme activities. This observation might be the reason why a high-fat diet is not adopted by the endurance athlete, although it has some merit in that there is an increase in mitochondrial enzyme activities and a concomitant decrease in utilization of glycogen during endurance exercise. Thus, the dietary regimen that induces increases in mitochondrial oxidative capacities in skeletal muscle without intra-abdominal fat accumulation and body weight gain will offer many advantages. In this context, the present study aimed to determine whether the repeated increase in FFA caused by an alternate-day high-fat diet results in an increase in the mitochondrial oxidative capacity without accumulation of intra-abdominal fat mass. Here, we report that an alternate-day high-fat diet, comprising a high-fat diet and standard diet every other day, has a significant effect on muscle mitochondrial enzymes—it increases mitochondrial enzyme activities and protein content without causing excess body weight gain and intra-abdominal fat accumulation.

## 2. Methods

### 2.1. Materials

Reagents for SDS-PAGE were obtained from Bio-Rad (Hercules, CA, USA). Monoclonal long-chain acyl CoA dehydrogenase antibody and horseradish peroxidase (HRP)-conjugated secondary antibodies were obtained from Sigma (St. Louis, MO, USA) and Cell Signaling Technologies (Danvers, MA, USA), respectively. Anti-PGC-1α antibody was obtained from Calbiochem (San Diego, CA, USA). Polyclonal antiserum specific for GLUT-4 was a generous gift from Mike Mueckler (Washington University, St. Louis, MO, USA). Enhanced chemiluminescence (ECL) reagent was purchased from Millipore (Temecula, CA, USA). All other chemicals were obtained from Sigma.

### 2.2. Treatment of Animals

Four-week-old male Wistar rats (70–90 g body weight) were obtained from CLEA Japan (Tokyo, Japan). All rats were housed in rooms lighted from 9:00 a.m. to 9:00 p.m. The room temperature was maintained at 22–24 °C. Rats were separated into those receiving a control diet (CON: *n* = 6), high-fat diet (HFD: *n* = 6), and an alternate-day high-fat diet (ALT: *n* = 6). The high-fat diet was prepared using lard, corn oil, sucrose, and casein (32%, 18%, 27%, and 23%, respectively, of total calories), supplemented with minerals (51 g/kg, AIN93G mineral mix: CLEA Japan), vitamins (22 g/kg, AIN93 vitamin mix: CLEA Japan), and methionine (4.4 g/kg: Wako Pure Chemical). The standard diet, CE-2 was obtained from CLEA Japan; it contained as percentage of calories, 59% carbohydrate, 12% fat, and 29% protein. The energy content of the high-fat diet was 5.1 kcal/g, whereas that of the standard diet was 3.4 kcal/g. The rats were provided with food and water *ad libitum*. Rats in the CON and HFD groups were fed the control diet and the high-fat diet for 4 weeks, respectively. Rats in the ALT group were fed the control diet alternated with the high-fat diet every other day. The ALT animals were fed the high-fat diet on the day before sacrifice. Food was removed at 9:00 p.m. the day before muscle dissection. Between 9:00 and 12:00 a.m. on the next day, rats were anesthetized with an intraperitoneal injection of pentobarbital sodium (50 mg/kg) and blood samples were drawn from the abdominal aorta. After the blood sampling, plantaris muscle and epididymal fat pads were removed. This experimental protocol was approved by the Committee for Animal Experimentation in the School of Sport Sciences at Waseda University (No. 2014-A096).

### 2.3. Measurement of Mitochondrial Enzyme Activities

For enzyme activity measurements, a portion of plantaris muscles were homogenized in ice-cold buffer containing 175 mM KCl, 10 mM GSH, and 2 mM EDTA, pH 7.4. The homogenates were frozen and thawed three times and mixed thoroughly before enzyme activities were measured. For the β-hydroxyacyl-CoA dehydrogenase (β-HAD) assay, an aliquot of the homogenate was centrifuged at 700× *g* for 10 min at 4 °C. Citrate synthase (CS), a marker of oxidative enzymes, and β-HAD activities were measured using Srere’s [17] and Bass’s [18] methods, respectively.

### 2.4. Western Blot Analysis

A portion of frozen plantaris muscles were homogenized in ice-cold RIPA buffer containing 50 mM Tris-HCl, pH 7.4, 150 mM NaCl, 0.25% deoxycholic acid, 1% NP-40, 1 mM EDTA, and a protease inhibitor cocktail (Cell Signaling Technologies, Danvers, CA, USA). Protein concentrations were measured using a BCA protein assay kit (Pierce, Rockford, IL, USA) according to the manufacturer’s instruction. Samples were diluted in 4× sample buffer (Invitrogen, Camarillo, CA, USA). Equal amounts of sample protein were subjected to SDS-PAGE (10% resolving gels) and then transferred to PVDF membranes at 200 mA for 90 min. After transfer, the membranes were washed in Tris-buffered saline with 0.1% Tween 20 (TBST; 20 mM Tris base, 137 mM NaCl, pH 7.6), and then membranes were blocked with TBST supplemented with 5% skimmed powdered milk for 1 h at room temperature. After blocking, the membranes were incubated overnight at 4 °C with antibodies specific for long chain acyl CoA dehydrogenase (LCAD), glucose transporter-4 (GLUT-4) and PGC-1α at concentrations of 1:2000–5000. The HRP-conjugated secondary antibody (goat anti-rabbit IgG) was used at a concentration of 1:10,000. Bands were visualized by ECL and scanned using a chemiluminescence detector (LAS 3000, FUJIFILM). The membranes were stained with Coomassie Brilliant Blue (CBB) to verify and normalize the protein loading [19]. Band intensities were quantified using ImageJ (NIH).

### 2.5. Analytical Procedure

Concentrations of plasma glucose, FFA, and triglyceride were determined using kits (Glucose C2 Test Wako, NEFA-C Test Wako, Triglyceride E Test Wako, respectively) according to the manufacturer’s instructions. Plasma insulin concentration was measured using an enzyme-linked immunospecific assay kit according to the manufacturer’s instruction (Mercodia AB, Uppsala, Sweden).

### 2.6. Succinate Dehydrogenase (SDH) Staining

For histological analysis, plantaris muscles were frozen in isopentane, which had been cooled in liquid nitrogen. Serial cross-sections (5 μm thick) were cut in a cryostat at −20 °C. Sections were stained for succinate dehydrogenase (SDH) activity, complex II of the mitochondrial respiratory chain, as follows. Sections were first allowed to reach room temperature before they were then incubated in a solution containing nitro blue tetrazolium (0.5 mg/mL), sodium succinate (50 mM), and phosphate buffer (0.12 M potassium dihydrogenphosphate, 0.88 M disodium hydrogen phosphate) for 25 min at 37 °C. Cross-sections were then washed three times in distilled water, dehydrated in 70% (1 min), 80% (1 min), 90% (1 min), and 100% (1 min) ethanol, and then cover-slipped using an aqueous mounting medium. 

### 2.7. Muscle Glycogen Concentration

Glycogen concentration in plantaris muscles was determined by using the method of Lowry and Passonneau [20] after acid hydrolysis.

### 2.8. Statistical Analysis

The data are presented as the mean ± standard error of the mean (SEM). Statistical analysis was performed using analysis of variance (ANOVA). The Tukey’s test was used for *post hoc* analysis when the ANOVA test indicated significant differences. When the normality (Shapiro–Wilk test) was not met, variables were analyzed using the Kruskal–Wallis test and the Steel–Dwass *post hoc* test was used as needed. Statistical significance accepted at *p* < 0.05.

## 3. Results

### 3.1. Body Weight, Epididymal Fat Weight, and Plasma Parameters

Table 1 shows the body weight and epididymal fat weight. The 4-week high-fat diet resulted in an increase in epididymal fat weight in the HFD group (CON *vs*. HFD, *p* < 0.05). However, at 4 weeks, epididymal fat weight in the ALT group was not significantly different from that in CON group.

Plasma FFA concentration in the HFD and ALT groups was significantly higher than that in the CON group (CON *vs.* HFD and ALT, *p* < 0.05). Although the precise mechanism is not clear, plasma glucose concentration in the ALT group was significantly lower than those of CON and HFD (ALT *vs.* CON and HFD, *p* < 0.05). There was no significant difference in plasma insulin concentration among the three groups (Table 1).

### 3.2. Mitochondrial Enzymes Activities and PGC-1α Protein Content

**Figure 1:**
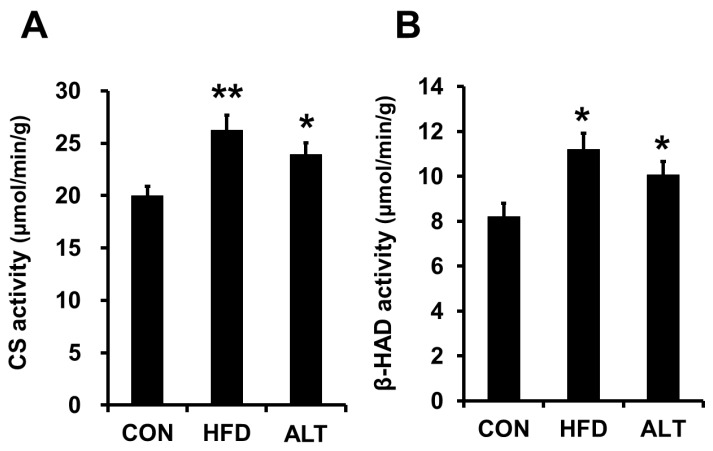
Effects of alternate-day high-fat diet feeding on citrate synthase (**A**) and β-HAD (**B**) enzyme activities in rat skeletal muscle. Values are mean ± SEM of 6 animals per group. * and ** indicate significant differences at levels of *p* < 0.05 and *p* < 0.01 *vs*. CON, respectively.

**Figure 2:**
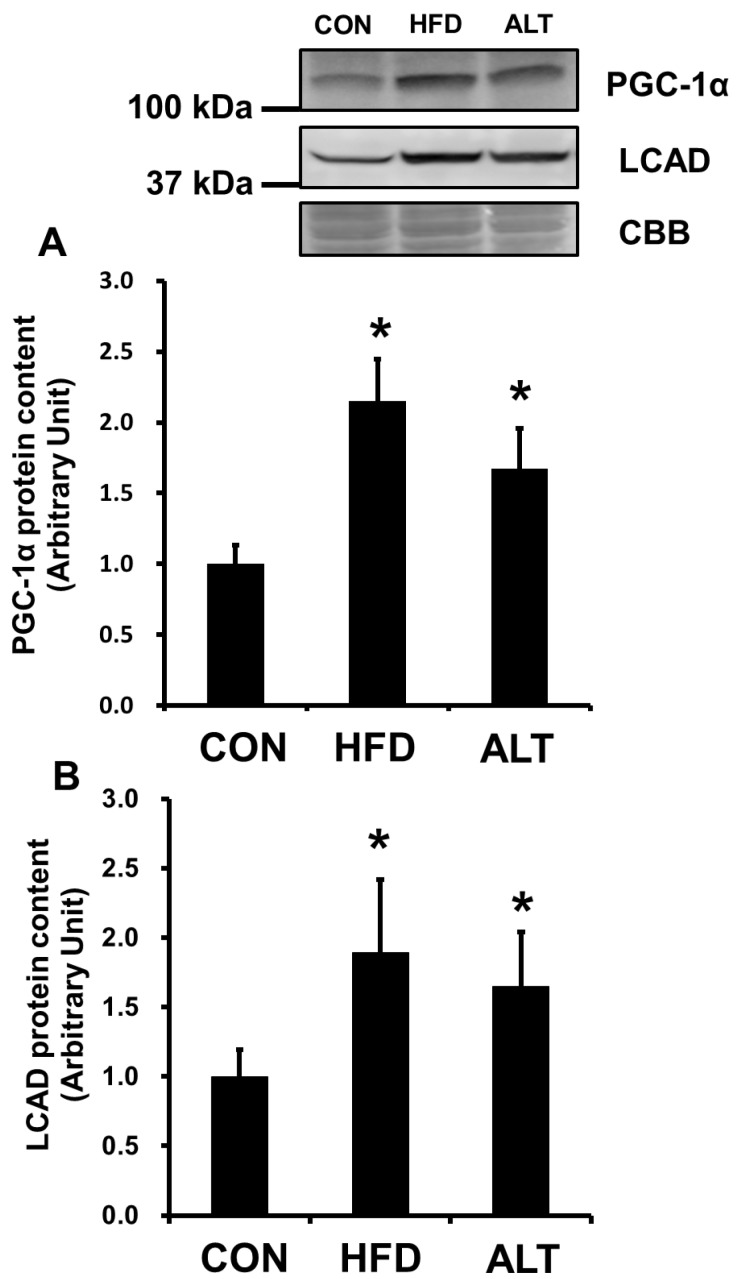
Effects of alternate-day high-fat diet feeding on PGC-1α (**A**) and LCAD (**B**) protein content in rat skeletal muscle. Values are mean ± SEM of 6 animals per group. * indicates significant difference at a level of *p* < 0.05 *vs*. CON.

Citrate synthase activities in the plantaris muscle of the HFD and ALT rats were significantly higher than in the same muscle of the CON rats (Figure 1A) (CON *vs.* HFD, *p* < 0.01; CON *vs.* ALT, *p* < 0.05). After the 4-week dietary intervention, the β-HAD activity in the HFD and ALT groups was significantly higher than that in the CON group (Figure 1B) (CON *vs.* HFD and ALT, *p* < 0.05). Protein content of PGC-1α in HFD group was significantly higher than that of the CON group (*p* < 0.05, Figure 2A). Furthermore, PGC-1α protein content was increased by 4-week alternate-day high-fat diet feeding (*p* < 0.05. Figure 2A). Both HFD and ALT induced a significant increase in LCAD protein content in plantaris muscle (Figure 2B) (CON *vs.* HFD and ALT, *p* < 0.05).

### 3.3. SDH Activity

**Figure 3:**
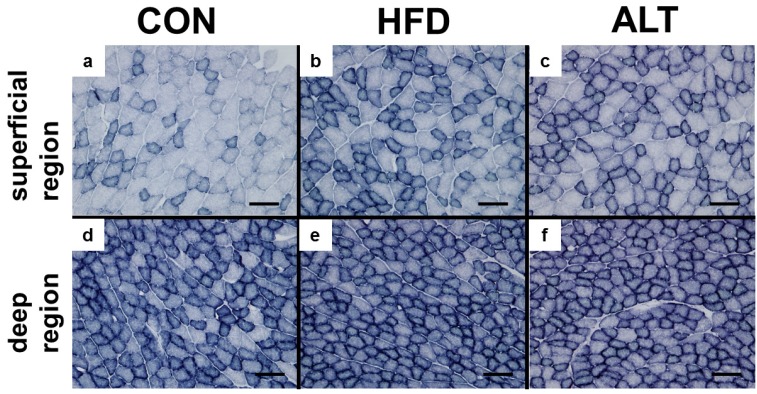
Effect of alternate-day high-fat diet feeding on succinate dehydrogenase (SDH) staining in plantaris muscle. Representative SDH-stained images are presented. SDH staining of superficial region of plantaris muscle from CON (**a**), HFD (**b**) and ALT (**c**) and deep region from CON (**d**), HFD (**e**) and ALT (**f**). Plantaris muscle of both HFD and ALT showed relatively dark staining for SDH compare to that of CON. Scale bar, 100 μm.

Next, we assessed the effect of an alternate-day high-fat diet on the oxidative capacity in skeletal muscles using histochemistry. Figure 3 shows representative images of SDH staining of the plantaris muscle from CON, HFD, and ALT groups. Succinate dehydrogenase activity staining was increased in HFD and ALT groups compared to the CON group.

### 3.4. Muscle Glycogen Concentration and Glucose Transporter-4 Protein Content

**Figure 4:**
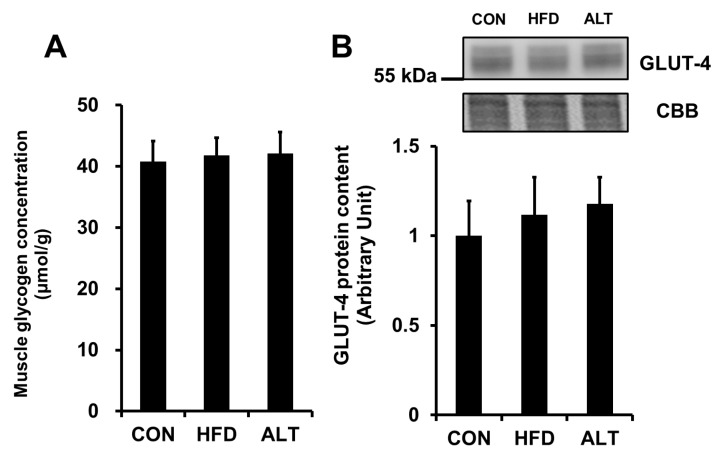
Effects of alternate-day high-fat diet feeding on glycogen concentration (**A**) and GLUT-4 protein content (**B**) in rat skeletal muscle. Values are mean ± SEM of 6 animals per group.

Previous studies reported that long term high-fat diet feeding reduces muscle glycogen concentration [4,6,8]. Since GLUT-4-mediated glucose transport across the plasma membrane is one of the rate-limiting step of glycogen synthesis in skeletal muscle [21], we measured muscle glycogen concentration and GLUT-4 protein content. As shown in Figure 4, we observed no significant difference in glycogen concentration (Figure 4A) or GLUT-4 content (Figure 4B) in plantaris muscles among the three groups.

## 4. Discussion

The main findings of the present study were that an alternate-day high-fat diet induces increases in mitochondrial enzyme activities and protein content in rat skeletal muscle, without causing intra-abdominal fat accumulation.

It was first reported by Holloszy [1] that endurance exercise training increases mitochondrial enzyme activities in rat skeletal muscle, and this finding was confirmed by other research groups assessing human skeletal muscle [22]. The most important physiological effect of an increase in mitochondrial content in skeletal muscle is the sparing of muscle glycogen during submaximal exercise. The glycogen-sparing effect mediated by a smaller decrease in creatine phosphate and ATP, and a smaller increase in inorganic phosphate, stimulates glycogenolysis [2,23]. Miller *et al.* [4] reported that rats, which were fed a high-fat diet for 5 weeks ran for a longer duration than those fed a high-carbohydrate diet. The improvement in endurance performance is concomitant with an increase in skeletal muscle citrate synthase (a key enzyme of the tricarboxylic acid cycle) and β-HAD enzyme activity (a major index of the β-oxidation) and lower utilization of muscle glycogen concentration. This result suggests that a high-fat diet induces an increase in mitochondrial biogenesis, muscle glycogen sparing during exercise, which thereby prolongs submaximal endurance exercise performance. In agreement with this finding, our results also showed that a 4-week high-fat diet induces an increase in key mitochondrial enzyme activities in rat skeletal muscle, and that an alternate-day high-fat diet induces an increase in mitochondrial enzymes in rat skeletal muscle to a level comparable to that observed after a daily high-fat diet (Figure 1A,B). This result suggests that an alternate-day high-fat diet is sufficient to increase mitochondrial enzyme activities and protein content in skeletal muscle. 

The main disadvantage of a long-term high-fat diet is the huge accumulation of intra-abdominal fat and increasing body weight. In the present study, intra-abdominal fat mass in HFD rats was about 60% higher than that of CON rats (Table 1). Since there is a strong correlation between intra-abdominal fat mass and insulin resistance [24,25], it is difficult to adopt the long term high-fat diet for endurance athletes. Not only is it particularly unhealthy, but also will it result in an increase in body weight, which negatively affects endurance exercise performance. However, an alternate-day high-fat diet induced muscle adaptation, but did not cause excessive intra-abdominal fat accumulation. Results from the present investigation suggest that it is possible that dietary intervention with a high-fat diet can induce increases in mitochondrial oxidative capacities in skeletal muscle, while reducing health risk. However, in the present study, the diet intervention period was only 4 weeks to determine the effect of alternate-day high-fat diet feeding on intra-abdominal fat accumulation. If a longer period of dietary intervention is performed, an increase in body fat might be observed. It should be investigated whether time course changes of accumulation of intra-abdominal fat by an alternate-day high-fat diet.

Recently, the possible mechanisms involved in this high-fat diet-induced increase in mitochondrial protein content in skeletal muscle have been studied. It has been reported that raising plasma FFA results in an increase in PPAR δ activation and mitochondrial biogenesis [13]. In this study, plasma concentration of FFA is higher in both HFD and ALT groups than in the CON group. Therefore, it is likely that there is a similar activation of PPARδ by raising plasma FFA in both the HFD and ALT groups resulting in an increase in mitochondrial enzyme activities in skeletal muscle. The transcriptional coactivator PGC-1α is known to induce mitochondrial biogenesis by activation of transcription factors and coordinated expression of a large number of proteins [26]. In this study, PGC-1α protein content in HFD was significantly higher than that of CON. Furthermore, the ALT group had an elevated protein content of PGC-1α (Figure 2A). It was reported that PGC-1α proteins significantly increase after 4 weeks of HFD, without an increase in the rate of transcription [13]. The finding that PGC-1α mRNA expression does not increase in skeletal muscle of rat fed a high-fat diet [13], suggested a hypothesis that high-fat diet results in an increase in PGC-1α protein content through post-translational mechanisms, such as decrease in degradation of PGC-1α protein. Although the precise mechanisms by which high-fat diet increases PGC-1α protein content through post-translational mechanisms are not clear, our findings suggested that repeated stimulation of HFD by ALT is sufficient to elevate PGC-1α protein content and mitochondrial proteins. 

Several studies reported that rats fed a high-fat diet are capable of intense exercise despite a limited muscle glycogen stores [4,6,8]. In the present study, we did not find differences in muscle glycogen concentration among the different groups. Furthermore, the protein content of GLUT-4, a predominant form of glucose transporter in skeletal muscle, was not different among the three groups (Figure 4A,B). The difference in glycogen concentration in response to a high-fat diet between our study and previous studies might be due to the fat content of the diet. The diet used in this study comprised 50% of calories from fat, whereas rats in the previous studies were fed diets comprising 78% fat [4,8]. This extremely low carbohydrate diet may result in lower glycogen concentrations in skeletal muscle.

In contrast to animal studies, human studies have failed to demonstrate a beneficial effect of a high-fat diet on endurance exercise performance [5,27]. The difference in results between animal and human studies may be because of fat composition in diet. The control diet used in most of the animal studies comprised approximately 10% of calories from fat, whereas a typical Japanese and American diet consists of about 25% [28] and 34% fat [29], respectively. It might be difficult to detect a high-fat diet-induced increase in endurance performance in humans, because the fat content in the diet of humans is higher than that used in experimental animals. However, because a high-fat diet, containing 62% calories from fat, induces increases in mitochondrial enzymes in human skeletal muscle [7], human skeletal muscle is capable of adaptation responses to a high-fat diet. Therefore, it will be interesting in future studies to determine whether manipulation of dietary fat, not using a high-fat diet, induces an increase in mitochondrial oxidative enzyme capacity in human skeletal muscle and enhance endurance exercise performance.

Recent works by Shortreed *et al.* demonstrated that high-fat diet feeding for 8 weeks impaired oxidative capacity in mice skeletal muscle [30]. In contrast, Sadler *et al.* reported that 2-week high-fat diet feeding in mice down-regulated citrate synthase activity, but it gradually increased at 16 weeks [31]. While it is difficult to be certain why the adaptations to high-fat diet differ from the studies, the time course of development of mitochondrial impairment by over-feeding or high-fat diet, and the differences of animal model (mouse or rat), should be investigated carefully in future studies. Furthermore, although the increase in mitochondrial volume, enzyme activity, and changes in organelle composition is referred to as mitochondrial biogenesis, we assessed the limited numbers of mitochondrial proteins, including β-oxidation enzymes (β-HAD and LCAD) and citrate cycle enzyme (citrate synthase) in this study. In addition, recent studies showed the functional role of mitochondrial reactive oxygen species, which affecting calcium handling proteins involved in muscle contractility [32,33]. Future extensive investigations are expected to directly measure the mtDNA copy number, mitochondrial volume, and calcium handling capacity of mitochondria to examine whether the alternate-day high-fat diet feeding induces an increase in functional mitochondria in skeletal muscle.

## 5. Conclusions

In conclusion, we found that provision of an alternate-day high-fat diet for 4 weeks induces increases in mitochondrial enzyme activities and protein content in rat skeletal muscle without intra-abdominal fat accumulation.

## Figures and Tables

**Table 1:** Effects of alternate-day high-fat diet feeding on body weight, epididymal fat mass, plasma glucose, free fatty acids, and insulin concentrations in rats.

	CON	HFD	ALT
Initial body weight (g)	87 ± 1	86 ± 5	87 ± 1
Final body weight (g)	298 ± 5	297 ± 9	298 ± 3
Epididymal fat mass (g)	3.1 ± 0.2	5.1 ± 0.3 *	3.8 ± 0.1
Plasma glucose (mg/mL)	96.9 ± 2.6	96.8 ± 6.3	81.1 ± 2.6 ^#^
Plasma FFA (mEq/L)	0.28 ± 0.02	0.44 ± 0.05 *	0.44 ± 0.06 *
Plasma insulin (µg/L)	0.39 ± 0.3	0.42 ± 0.4	0.39 ± 0.3

CON, control group; HFD, high-fat diet group; ALT, alternate-day high-fat diet group. Values are mean ± SEM of 6 animals per group. * indicates significant difference at a level of *p* < 0.05 *vs.* CON. ^#^ indicates significant difference at a level of *p* < 0.05 *vs*. CON and HFD.
